# Sustainable manufacturing through application of reconfigurable and intelligent systems in production processes: a system perspective

**DOI:** 10.1038/s41598-023-49727-5

**Published:** 2023-12-16

**Authors:** Marco Todescato, Orjola Braholli, Dmitry Chaltsev, Ilaria Di Blasio, Davide Don, Georg Egger, Julius Emig, Gabriele Pasetti Monizza, Pasqualina Sacco, Dietmar Siegele, Dieter Steiner, Michael Terzer, Michael Riedl, Andrea Giusti, Dominik Matt

**Affiliations:** 1 Fraunhofer Italia Research, Bolzano, 39100 Italy; 2 Free University Bolzano, piazza Università 1, 39100 Bolzano, Italy

**Keywords:** Mechanical engineering, Computer science, Information technology, Software

## Abstract

Sustainable production aims at creating products from processes that minimize environmental impact, energy consumption and natural resources. Customers and markets are ever more leaning towards digital, custom, and flexible solutions with lower environmental impact. Hence, Industry 4.0 (I4.0) solutions are increasingly including social and environmental sustainability aspects. We focus on the realization of an infrastructure integrating industrially relevant application modules by combining system reconfigurability and artificial intelligence, towards sustainable production. To meet the final goal of sustainable production, we address four challenges considering flexibility and sustainability in production in a holistic way: (1) developing infrastructural and methodological tools to support companies to explore the potential of I4.0 towards sustainable production; (2) managing the configurability and customization possibilities of products; (3) effectively handling the flexibility provided by a production system with rapid reconfiguration capabilities; (4) integrating hardware and software flexibility by using reconfigurable robotics and machine learning methods. By developing and connecting different application modules, we obtain a physical demonstrator which represents on the one hand an exemplary scenario of reconfigurable and flexible production system; on the other, it enables new research activities and insights with a *see, touch & feel* approach for industrial and research realities.

## Introduction

Sustainable manufacturing aims to create products based on processes that minimize negative environmental impact, preserve energy and natural resources, are safe for employees, the community, customers and are economically sound^1^. Market preferences are increasingly oriented towards product solutions that are customizable, flexible, and have the least impact on the environment. In this context, the benefits of the applications of Industry 4.0 (I4.0) can be associated with aspects of sustainability, both social and environmental^2^. Sustainability is usually described in its three main dimensions (or pillars)—social, economical and environmental—which, despite all three being fundamental when dealing with production, should also be considered from a broader perspective. For instance, as reported by the International Organization for Economic Cooperation and Development (OECD)^3^, it is paramount to consider the relevance that sustainable practices represent in the eyes of numerous stakeholders such as investors, regulators, customers and of the communities in which companies (including Small and Medium Enterprises—SMEs) operate. In particular, OECD observations indicate that (1) financial analysts rate companies with good environmental reputations better than others, that (2) by simply reducing energy consumption, a 5% increase in overall profits can be achieved, and finally (3) as reiterated by a survey carried out on $>5000$ subjects on a global scale^4^, young workers demands jobs that are active or conscious to sustainable topics.

Manufacturing systems will be called upon to adapt, at an ever increasing rate, to demands for changes in products, processes and technologies used. These adaptation needs are usually imposed by lack of resources, strict regulations and highly volatile market demand^5^. Classical manufacturing systems, such as dedicated production lines, are based on rigid automation and designed to handle a single product, or few pre-determined variants, at a high production rate, failing to adjust to highly variable environments. Classical manufacturing systems are designed—and possibly optimized—to perform specific tasks over long periods of time, to achieve a return on investment and thus be economically viable in the long run. However, production optimization based on the assumption of demand for the same type of product clashes with current trends which are shifting from mass production to mass customization (MC). Such a clash can be a strong disincentive for SMEs to invest in automation systems if this could undermine the ability to adapt quickly to market demands thus reducing their flexibility.

Defined by Davis^6^ as the capability of manufacturing tailored products with production costs like those of mass-produced products^7^, MC allows each customer to configure a product through a set of predefined features to design her/his own individualized product, fulfilling her/his specific needs^8,9^. Since its first formulation, MC has received a growing consideration by the scientific community both in manufacturing and in service delivery^10^. Despite in its general meaning customization sounds the opposite of standardization and modularization, Krstić et al.^11^ highlight they are complementary parts of a production business. Indeed, their study highlights that a production concept should be defined based on a synthesis and on a synergy of standardization and modularization (of standard modules) to deliver product customization, according to the specific needs of the addressed market target^11^. Also, a recent survey from the McKinsey Institute^12^ reported that 71% of consumers expect companies to deliver customized interactions with 76% getting frustrated when this doesn’t happen. Interest in customization is growing ever faster and the scientific community is focusing on application of mass customization approaches in modern smart manufacturing environments. However, MC brings radical changes to methods used to operate traditional manufacturing systems^13,14^ as production systems in a mass customization environment target to produce small quantities in a highly flexible way and to be rapidly reconfigurable^9,15^.

A promising avenue for the realization of this production scenario is to incentivize and establish the use of approaches of highly flexible automation that introduce capabilities of rapid re-configurability and re-programmability, with the highest possible degree of achievable autonomy. It is foreseen that intelligent and self-optimizing manufacturing systems will learn and there-by perform self-determined changes in production systems^16^. To reach such a next level of changeability it is necessary to equip manufacturing systems with capabilities to take autonomous decisions in even more complex production processes with a high product variety^17^.

**Figure 1:**
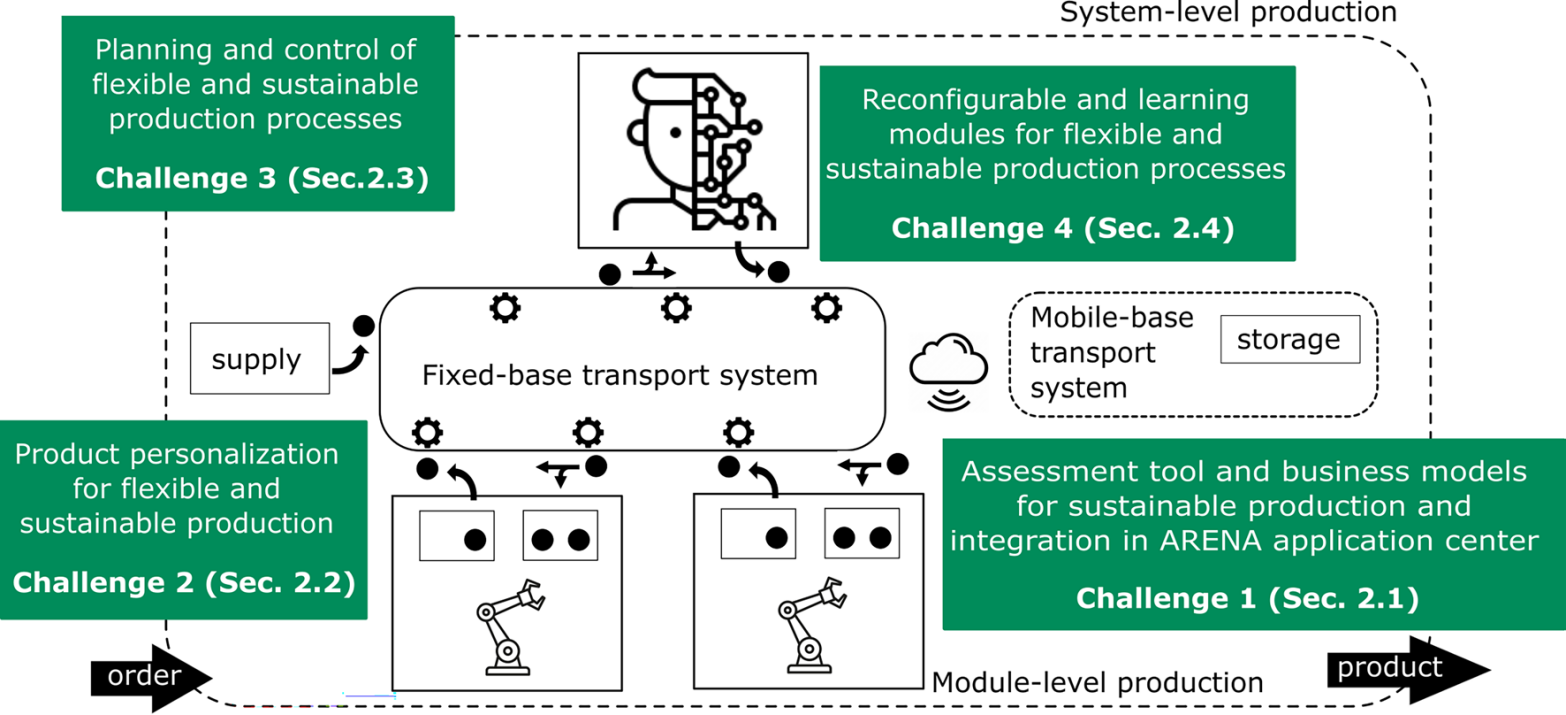
Challenges for smart and sustainable manufacturing in production processes.

Given the above context, this manuscript aims to provide an approach, that considers flexibility and sustainability holistically, to move from mass production to mass customization manufacturing systems while alleviating the radical changes due to this paradigm shift^13,14^. The research questions we want to address towards this goal are: (1) are companies able to quickly assess their potential benefit from the exploitation of I4.0 technologies also in view of sustainability? (2) Do customers have the possibility to really participate in customizing their desired products while actively considering sustainability aspects? (3) How can automation, automatization and the recent advancements of information technologies and artificial intelligence play the main character role in a new era of flexible and sustainable manufacturing? To tackle such questions, with reference to Fig. 1, a prototypical manufacturing system has been broken down into its core building blocks^18^ from product design to delivery. Focusing on those strictly related to the manufacturing process, i.e., *product design*, *manufacturing infrastructure*, and *production modules*, despite several unrelated solutions that address several of the aforementioned aspects can be found in the literature from different fields, we propose how to seamlessly integrate them into a physical demonstrator. In contrast to classical manufacturing systems, our proposed demonstrator indeed allows to showcase and address flexibility and sustainability from a holistic perspective. In particular, we identify and address four *main challenges*, also shown in Fig. 1, whose solutions lay the necessary building blocks for flexible and sustainable manufacturing. They aim at the development of infrastructural and methodological tools: (1) to support companies to explore the potential of I4.0 towards sustainable production; (2) to manage products configurability and customization possibilities; (3) to effectively handle the flexibility provided by a production system with rapid reconfiguration capabilities; (4) to integrate hardware and software flexibility by using reconfigurable robotics and machine learning methods. In addressing them, it will be made clear how each challenge relates to the above research questions. Ultimately, our demonstrator shows how possibilities for rapid adaptation can be exploited in favor of criteria that lead to the achievement of sustainable production. In addition, this paper presents the methodological aspects used to address the main challenges and it shows how methodology is grounded into the proposed physical demonstrator, resulting in a production-system perspective of sustainable manufacturing.

The remainder of the paper is organized as follows: section “Methods” covers methodological aspects addressing the above mentioned four main challenges (illustrated in Fig. 1) each of which presented in one subsection. Section “Results” presents the results and their integration into our physical demonstrator. Section “Discussion and conclusion” discusses the results and concludes the manuscript.

## Methods

### Assessment tools and business models for sustainable production

First introduced in the nineties, sustainable production is not a novel concept^21,22^. J. Elkington encouraged the “Triple Bottom Line” (also referred to as the “Triple P” framework: People, Planet and Profit) which integrates economical aspects, typical of the economic management of industries, with the environmental and social dimensions in an unified framework. According to Elkington, by incorporating this multidimensionality in the business model it is possible to improve the quality and sustainability of delivered products, the impacts of the whole production and post-consumer phase while ensuring the growth of the company. Nonetheless, as of today, companies sometimes have not a complete picture of their current state and how to implement a more sustainable strategy.

**Figure 2:**
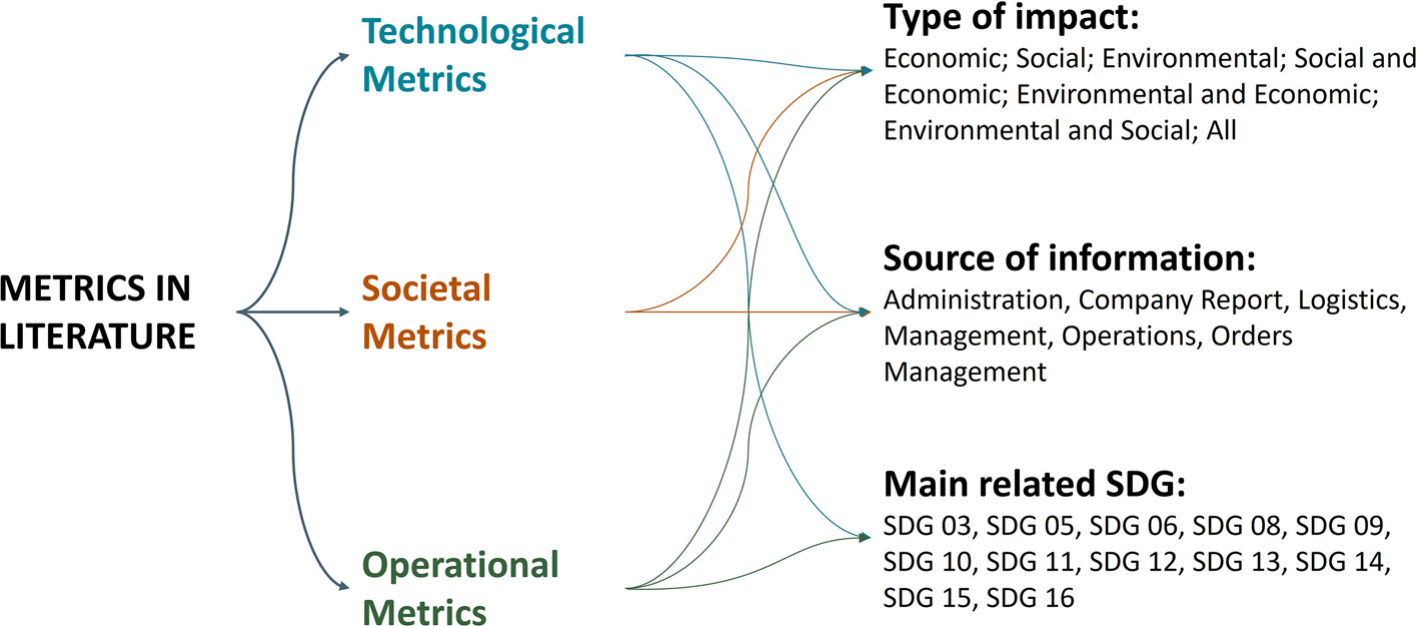
Sustainability measure categorization and clustering according to different dimensions and criteria. Sustainability Development Goals (SDG) available from the Agenda 2030 for Sustainable Development^19^. Figure adopted and adapted from Don et al.^20^.

The first challenge of this work aims to directly tackle the first formulated research question, i.e., *are companies able to quickly assess their potential benefit from the exploitation of I4.0 technologies also in view of sustainability?* To deliver an assessment tool to allow companies to quickly and easily evaluate their positioning within the three sustainability pillars, our approach builds upon the framework first presented by Don et al.^20^. The framework is in turn based on a multi-criteria categorization and clustering of several metrics and indicators, generically referred to as measures, which allow users to better explore and capture how each single measure affects and is involved with different sustainability dimensions.

Don et al.^20^ highlight on the one hand that for most of the collected measures from the literature the focus is on efficiency of one or some parameters; on the other however, that there is evidence that the impact of a parameter should be evaluated by looking at more than one sustainability pillar. Indeed, an indicator could produce benefit to more than one pillar or conversely, create a benefit for one while producing a cost for the other. Moreover, assessing whether a measure affects more than one sustainability dimension enables to approach the problem from an holistic perspective and thus to be able to decide which are the meaningful and actually applicable parameters for an organization. Figure 2 illustrates the categorization and clustering each measure under analysis is subject to, while, in section “Results” we described how a subset of selected measures have been grounded in our physical demonstrator.

### Product personalization for flexible and sustainable production

By definition, mass customization production environments need to produce small batches of products flexibly and rapidly. Ideally, customers should be given the possibility to freely customize their products, in light of the system manufacturing capabilities, with the system directly translating customization requests into technical specification. Thus virtually closing the physical gap between configuration and production. Considering sustainability as an additional factor, customers should also be made aware of how their requests impact this aspect, in order to take informed decisions.

In light of this, the second challenge considered contributes to the second formulated research question, i.e., *do customers have the possibility to really participate in customizing their desired products while actively considering sustainability aspects?* We address it through the development of a Smart Product Configuration System (SPCS). Indeed product configurators aim at overcoming the gap between customer needs and manufacturing capabilities—both in business-to-business (B2B) and business-to-consumer (B2C) relationships—by relying on different approaches: rule-based, model-based, and case-based^23,24^. Multiple research approaches explore the adoption of different computational algorithms for delivering a product configuration: from multi-objective frameworks^25–27^ to fuzzy logic^28^ as well as in the form of constraint satisfaction problems^29,30^. The challenge of mass customization and the adoption of advanced computational methods, e.g., deep learning techniques, highlight a semantic gap between customers and suppliers (especially in B2C relationships) because customers may not have enough expertise about unfamiliar products^31^. To solve this semantic gap, Randall et al.^31^ introduce the concept of needs-based system which aims at translating natural language in product specifications. However, in Engineer-to-Order (ETO) industries^32^, the customer order decoupling point is located very early in the value chain, which means that design and engineering are part of the value chain system^33^. This implies that, beyond semantic description of specification, product design and engineering must solve specific customer needs. Accordingly, in this work, we explore the integration of product design and engineering in early product configuration steps through an SPCS with a twofold nature: on the one hand, to provide through its front-end, a graphical user interface (GUI) for users to customize a desired product and request their production to the system, while receiving sustainability information as feedback from the system; on the other, through its back-end, to be part of the Multi-Agent System (see section “Planning and control of flexible and sustainable production processes”) in order to interface with later production processes to trigger a scheduling management of the manufacturing system. With this approach, we investigate possible data automation methods in complex environments such as ETO industries, trying to reduce the distance between customer and production system.

Focusing on the overall functional user experience, a pre-defined set of prototypical standard parts has been introduced (detailed next in section “Results”). We consider a product as a combination of standard and “free-form” customizable parts. This combination allows to test degrees of standardization, modularization, and customization, both in product design and in product manufacturing.

The SPCS allows users to input a desired product configuration and, trigger its production through standard functionalities, such as: *select* a standard component from a pre-engineered library of objects, *place, move, and rotate* a standard component within an assembly space, *defining* a custom component through a free-form definition, *set* standard jointing elements and, *send* the product configuration to a production scheduling system or to a production management system. Also, the SPCS delivers data coming from other functional components of the manufacturing system, such as: production time, resource availability and sustainability-related indicators. Besides providing a preview of a product configuration, these data can be used proactively to deliver specific suggestions on possible actions to take, e.g. optimize predefined metrics.

The interaction between users and the SPCS relies on two different interfaces: (1) *hardware input devices*, such as touchscreen, VR headset, navigation mouses, or leap motion, and (2) *software input data collectors* such as graphical user interfaces (GUIs) specifically designed for each hardware input device.

To maximize the coverage of potential users and, at the same time, to deliver a flexible solution interacting with multiple functional layers within a company, we realized two versions of SPCS, according to two different user profiles:

1. **Computational CAD (CCAD) configurator**: based on a computer-aided design (CAD) system and developed to deliver a geometric abstraction of the product to a professional use, e.g., a product engineer or a designer.
2. **eXtended Reality (XR) configurator**: based on a game-engine system and developed to deliver a product simulation to a final user, such as a client.

**Figure 3:**
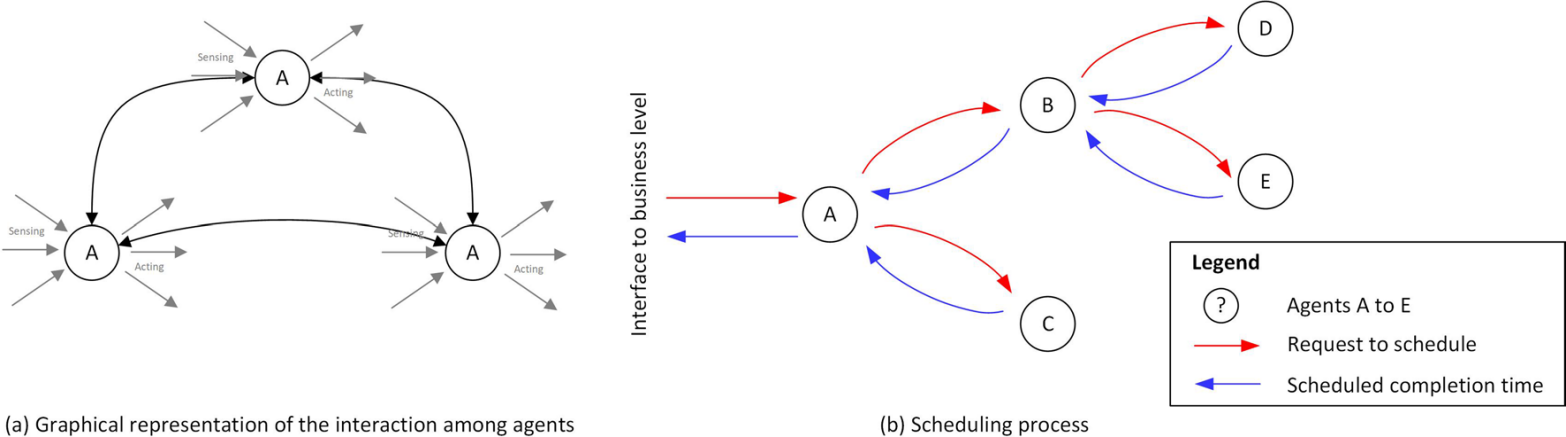
The MAS propagates initial requests towards the leaf nodes. Non-leaf nodes receive answers from sub-sequent agents with information about scheduled completion time in order to plan their own execution.

### Planning and control of flexible and sustainable production processes

After product configuration, a fundamental brick of production systems is concerned with planning and execution of production processes. Manufacturing Planning Systems (MPS) are concerned with planning of material and resources and, as such, can be integrated with Enterprise Resource Planning and usually operate on long (sector-dependent) time scales (from days to months)^34^. Manufacturing Execution Systems (MES) on the other hand control relevant modules of a production line and must be able to react within short time to unforeseen events^35^. Traditionally, manufacturing facilities are controlled by strictly hierarchical monolithic MESs following the ANSI/ISA 95 and IEC 62264 architecture, typically only loosely interconnected with the MPS (often through human actions only)^36^. The advantage is that of controlling all relevant data and operations globally with the drawback of a complex system to maintain and test^37^.

In view of this traditional paradigm, the third challenge in concerned with effectively handling the flexibility provided by a production system with rapid reconfiguration capabilities, in turn giving contributing to answer to the third research question, i.e., *how can automation, automatization and the recent advancements of information technologies and artificial intelligence play the main character role in a new era of flexible and sustainable manufacturing?*

When looking to optimize the development and maintenance efforts of these systems, an obvious option is to eliminate the division between MPS and MES and to unify their functionalities in a system that has both the near real-time characteristics of a MES and the overview to plan well ahead of time under global business-driven optimization criteria (e.g. machine utilisation)^38,39^. To achieve this and to maximize system modularity and reconfigurability, we propose a multi-agent solution (see Fig. 3a) which builds upon and expands the previous work by Egger et al.^40^ to embed sustainability aspects during planning and control. The proposed solution is based on a fully distributed architecture where intelligent actors can participate to the overall manufacturing capabilities, thus contributing to flexibility at a *system-level*, in a plug-n-play manner (provided they meet a commonly defined interface). This in order to make a step forward towards the problem of flexible *lot-size-one* production which in turn requires rapid reconfiguration of production lines as well as the ability to continuously integrate new production skills.

In the context of I4.0, a MAS serves the purpose of connecting multiple software entities and equipping them with intelligence and communication functionalities^35^. In particular, the term *agent* refers to a software program implementing a two-fold nature: on the one hand, it operates a physical production machine by e.g., sending/receiving machine signals and querying machines about expected task durations; on the other hand, it represents the machine in the cyber network of agents by, e.g., receiving/interpreting messages coming from others, scheduling tasks for the machine, notifying other agents about job durations, completion, or delays.

**Table 1:** Ontology used for system and product definitions.

production module	The physical resource (controlled by a production agent) providing specific capabilities to the factory.
production agent	A production module represented in the MAS by an agent, and that is in charge of management of the resources
order	Identified by a unique ID, it contains order items, and attributes like customer, order date, etc.
order item	Specific to a product type and to the ordered amount. It can include attributes like unit price, overall price, customized parameters, and priority.
product-type	Representative for a specific group of products that share the same production plan.
product-type graph	Hierarchical description, in form of a graph, of the physical composition of a product type without including information about how it is produced.
production plan	Template of operations and their relationships describing how a product type is manufactured. Conveniently represented as a graph.
operation	Action executed by production modules.
production item	At scheduling time, every piece of the order item is transferred to a production item that represents the individual product (quantity=1).
component	Atomic parts which a product is made of. Identified by a set of attributes such as name, type and material, geometry.

In this work an entire production process, i.e., from product configuration to delivery, the MAS is specifically in charge of (1) connecting all agents and, (2) planning and execution management of the physical production. More specifically, with reference to the ontology in Table 1 that defines useful production concepts, a production process consists of the following series of operations:

1. a user designs its new product-type thanks to a SPCS (if the product is already available in a library, it selects it), based on personal and/or functional preferences and, whenever appropriate and available, being informed on critical data, e.g., delivery time, sustainability-related parameters, amount of production resources;
2. in the background, whenever a new product-type is created, a corresponding product-type graph is generated and stored;
3. a user creates an order, possibly consisting of multiple product-types, amounts and additional relevant information (price, penalties for not finishing within a desired time, etc.)
4. the order is received by an *Order Agent*, responsible for translating the order into a production plan (if a plan is not already available)
5. the plan is scheduled, executed and controlled by the production agents in the MAS.

During step 5 agents schedule production based on the physical modules they are in charge of. Specifically, a production module might include different machines: typically, a principal one defining its main functionality (e.g., laser cutting), plus auxiliary ones (e.g., indicator lights). Following Egger et al.^40^, agents schedule production tasks according to a message passing mechanism where messages are encoded as JSON strings defined according to FIPA protocols^41^. In particular, upon receiving a request to perform a certain task agents might do one of the following (see Fig. 3b for an illustration):

- if a task is a leaf-node task (i.e. a node of the production plan with no additional following dependencies), the agent finds and reserves the first possible time slot when the requested task could be scheduled, and then answers to the requesting agent with the time of planned completion for the task;
- if a task corresponds to a non-leaf node, i.e. a node with children nodes, a request is sent to all agents in order to find one capable of performing the tasks. The own task is scheduled in available time slots, considering the completion times from the dependent tasks, ensuring the precedence constraints from the production plan.

The above scheduling procedure ensures coherent timing across the agents. Also, the system can react to events, by continuously communicating with the agents. Finally, observe that during the scheduling process, upon each transmission, the transmitting agent receives proposal responses from all the agents capable of performing the request tasks. Each proposal is evaluated by the agent according to certain boundary conditions and fitness functions and the best is selected. More specifically, such conditions consist of *hard constraints* such as latest acceptable completion time and resource availability, as well as *soft (optimization) constraints* such as different sustainability metrics (see section “Assessment tools and business models for sustainable production”).

Section “Results” introduces all the agents implemented in our physical demonstrator together with their roles in the production process and in the MAS.

### Reconfigurable and learning modules for flexible and sustainable production processes

The fourth and last challenge is also related to the third research question, i.e., *how can automation, automatization and the recent advancements of information technologies and artificial intelligence play the main character role in a new era of flexible and sustainable manufacturing?* However, differently from the third challenge of section “Planning and control of flexible and sustainable production processes”, it aims at providing a solution to *module-level* reconfigurability of manufacturing systems. Indeed, we target to provide a paramount feature of a flexible production system which consists in its ability to quickly adapt to new processes and products in order to meet custom demands. In view of this, the fourth and last major challenge we consider is to provide production modules which: (1) are easily reconfigurable (both at hardware and software level) to make them accessible also to non-professional users; and (2) that are capable of accounting for different sustainability aspects at production level. To meet these two objectives with our proposed demonstrator, we consider two complementary production modules, namely the *Human-to-Machine* (H2M) transfer manual assembly station and the *Modular Robotic Module* (MRM). The former module is used to extract knowledge from manual assemblies which are then transferred to the latter module to be executed automatically.

#### Human-to-Machine transfer (H2M) manual assembly station

Assembly tasks are ubiquitous in production processes. However, despite the possibility to perform predefined and repetitive assembly operations (in structured environments) thanks to custom designed and meticulously controlled robots, fine-grained dexterous assemblies still require human operators. Nonetheless, an ever larger portion of robotics research focuses on extracting and transferring knowledge from human experience to robots^42–44^. Within this context, we realize a production module characterized by a three-fold nature. First, to be a standard manual assembly station, consisting of off-the-shelf industry available standard solutions. Second, to support non-invasive data collection of human operations, i.e., introducing minor modifications and efforts to standard assembly processes of an operator. Third, to implement knowledge extraction and transfer of the experience gathered during human operations to synthetize automated assembly processes for dedicated robotic assembly modules. As better described in Sec. 3.4, to meet goals and constraints, the H2M station essentially consists of an off-the-shelf workstation for manual assembly, enhanced by an optical sensor system to track manual assembly operations. The tracked motions from the manual assembly operations are in turn used to learn suitable statistical models for H2M skill transfer.

#### Modular robotic module (MRM) for automatic assembly tasks

Traditional robot manipulators have a fixed structure, i.e., number of joints and links, geometry etc., which in turn defines the robot workspace. Fixed-structure manipulators happen to be “wrongly-sized” if used in multiple applications. They might be bulky with an eccessively large/small workspace or unnecessary number of degrees of freedom (DoF). In view of this, reconfigurable robotic platforms consist, by hardware design, of multiple types of modules that can be assembled into different structures to be able to cover for workspaces of different size and a variable number of DoF^45^. The major advantage is the possibility to adjust the robot structure to best match a given task while fulfilling and/or optimizing for additional constraints such as production time or energy consumption^46,47^. As better presented in section “Assembly and production”, by building upon a recently developed proposed reconfigurable system, we include a *modular robotic module* to address hardware flexibility during assembly operations.

**Figure 4:**
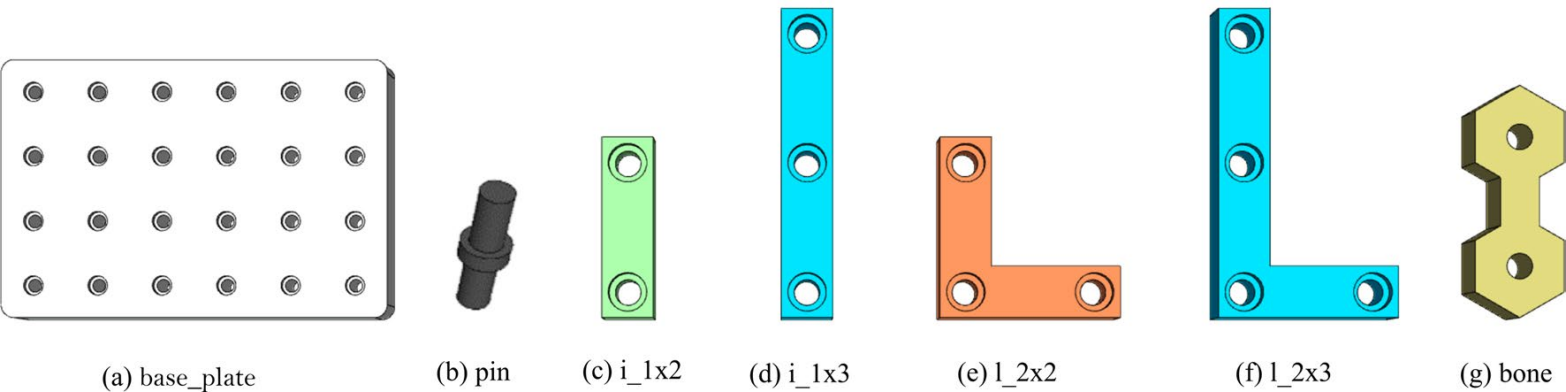
Illustration of the component used during product production.

**Table 2:** List of standard product components and an example of a “free-form” custom one.

Name	Type	Material	Mass [g]	CO$_2$ emiss. [gCO$_2^{eq}$]	Notes
base_plate	base plate	plastic	NA		Carrier board to facilitate product assembly
pin	joint	plastic	NA		Connection element between product components
i_1x2	standard	plastic	6.22	2.74	Short vertical standard component
		plywood	5.60	0.89	
i_1x3	standard	plastic	11.06	4.87	Long vertical standard component
		plywood	9.51	1.52	
l_2x2	standard	plastic	10.58	4.66	Small angular standard component
		plywood	9.70	1.55	
l_2x3	standard	Plastic	14.58	6.42	Elongated angular standard component
		Plywood	13.43	2.14	
Example bone	Custom	Plywood	NA		Random example of a custom component

## Results

The approaches to address the above mentioned challenges have been combined and grounded into a physical demonstrator available at Fraunhofer Italia’s *Area for REsearch and iNnovative Applications* (ARENA)^48^. The resulting demonstrator represents an essential yet complete example of flexible and reconfigurable manufacturing system. The demonstrator is the outcome of the integration of a set of different production modules whose ensemble addresses the four challenges presented in section “Methods”.

### Standard components & prototypical products

Production items consist of a combination of standard and custom components. Specifically, the components considered in the proposed demonstrator are showed in Fig. 4 and described in Table 2 which reports also data about materials, mass and equivalent carbon emission to produce each component. In particular, of the various metrics identified by the sustainability framework represented in Fig. 2, these three are deemed most suitable for integration in our demonstrator as they all fall onto the technological macro-category, have a direct environmental and economical impact and mainly relate to the Sustainability Development Goals 12 and 13 set by the Agenda 2030^19^. Such data are used within the sustainability framework to compute total carbon emissions associated to a configured product in order to notify the user and, if possible, suggest him/her with better options. Please note that some components data are listed as NA either because they cannot be precomputed, as for custom parts, or because they are not considered as part of the final product but just as support material used in the assembly process and thus reusable.

Thanks to the components listed in Table 2, the demonstrator is able to showcase different reconfigurability and sustainability aspects e.g., by exploring how product materials impact certain sustainability metrics which are reported to the user thanks to the configurator user interface.

**Figure 5:**
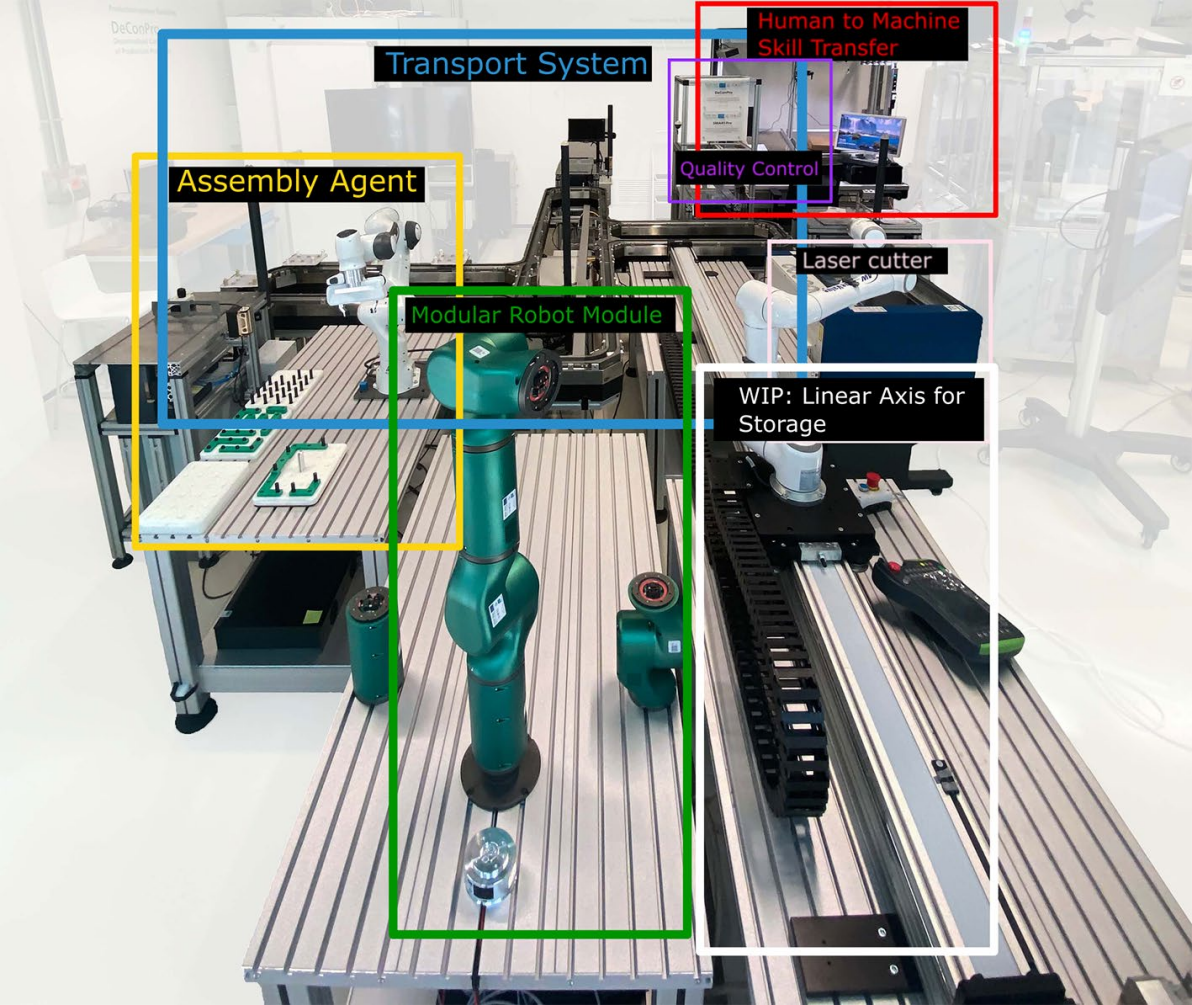
(better view in color) The proposed demonstrator. Highlighted with colors are different production modules.

### Composition of the demonstrator

The physical demonstrator is showed in Fig. 5. It consists of different physical modules, highlighted with colors, each of which in charge of different production tasks. These are:

- transport System module (blue) that is the physical interface to connect multiple production modules in the demonstrator;
- quality Control module (purple), used to inspect products after production and before delivery;
- laser Cutter module (pink) that is a production module to cut custom components made of plywood;
- human to machine skill transfer module (red) used for manual assembly and that implements learning from demonstration for assembly processes (see section “Reconfigurable and learning modules for flexible and sustainable production processes”);
- modular robot module (green), in charge of assembly functionalities and characterized by reconfigurability features (see section “Reconfigurable and learning modules for flexible and sustainable production processes”);
- assembly module (orange), similar to the Modular Robot, that is a robotic manipulator equipped with assembly functionalities of standard component;
- linear axis (white) equipped with an additional robotic arm to consider as an automatic storage for the system.

In addition, not shown in the picture, the demonstrator contains the *Product Configurator* module, which, as explained in section “Product personalization for flexible and sustainable production” and also described in section “Product configurator(s)”, aims at interfacing different types of users to the system, allowing them to customize their own products while communicating/visualizing relevant production/sustainability information. For each module, dedicated software agents (according to the multi-agent framework presented in section “Planning and control of flexible and sustainable production processes”) are implemented in order to make each module functionality available to the MAS. Additionally, a pure software-based *Order Agent*, is in charge of receiving orders submitted from the Product Configurator, creating the production plan and starting the production scheduling process.

In what follows, we outline how different modules/agents are practically implemented as well as how they address the challenges of section “Methods”.

### Product configurator(s)

#### CCAD configurator

The CCAD configurator offers a computational design tool within a CAD environment. The latter provides a geometric description of the product following standard conventions of technical drawings. The CCAD configurator consists of three main functional components:

- it develops the *product-configuration logic* which defines the base functionalities outlined in section “Product personalization for flexible and sustainable production” by exploiting internal functions of the CAD environment while enhancing standardization, modularization, and customization capabilities of the SPCS;
- it provides a *Graphical User Interface (GUI)* which offers a front-end interface to facilitate user interaction with the product-configuration logic;
- it implements a *computational design algorithm* which handles start-up imports, objects/components placements, material selection, collision/clash detection among placed components.

**Figure 6:**
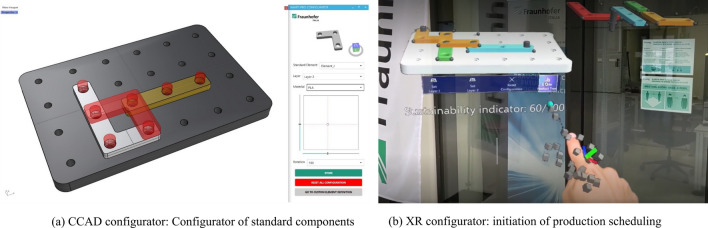
Screenshots of the two smart product configuration systems (SPCSs) implemented in our demonstrator.

After start-up imports, a user is prompted to select and place the desired amount of predefined standard components. In particular, for each component, the user can select its material (if multiple are available) and place it (according to discretized translations and rotations and arranged on two vertical layers) within a pre-loaded base_plate which defines the allowed assembly space (see Fig. 6a). After every positioning, a collision detection algorithm checks whether the new component can be placed or not. Once all the standard components are placed, users are prompted to draw free-form custom components thanks to a dedicated drawing tool. As soon as a close polyline is drawn, users can store the desired component, while the configurator, on the one hand, automatically modifies the desired free-form component to a final geometry which avoids intersections with standard components already on the assembly space; on the other, it places all the necessary pins for jointing purposes. Finally, if successful, the product features are stored in a product tree while a non-editable 3D geometry of the product is fixated within the assembly space. At this point, the product is ready to be sent to production after conversion to a suitable .json file.

#### XR configurator

The XR configurator allows us to create a product configuration by implementing an eXtended Reality experience. To accomplish this, the developed solution builds upon multiple software stacks which combine 3D modeling, real-time simulation and mixed-reality tools. In particular, a 3D CAD software (Rhinoceros 3D^49^) has been used to build the 3D models of the product components, in turn needed to generate XR interactive holograms, which are imported into Unity Real-Time Development Platform (Unity)^50^ for real-time simulation. Unity is indeed used to create the XR environment following a two-steps approach. The first step involves the integration of the Microsoft Mixed Reality Toolkit (MRTK)^51^ into Unity to facilitate the development of XR applications by providing functionalities for object manipulation and graphical user interfacing. Specifically, the following list is used to interact with standard product components:

- NearInteractionGrabbable: allows object grabbing at near distance;
- ConstraintManager: constraints object manipulation by allowing only certain transformations;
- ObjectManipulator: enables manipulation with XR-devices and translation and rotation using one or two hands;
- BoundsControl: facilitates interaction by displaying boundaries and handles for object manipulation.

In addition, thanks to ButtonCollections, four buttons are created to embed functionalities not directly related to object manipulation. The second step involves the implementation of a set of customized functionalities in order to provide a better customer experience. This is achieved integrating custom-developed functions into the MRTK ObjectManipulator interface that allow specific behaviors (e.g., keep track of object positions, discrete rotation, snapping) during interaction with an object. Similarly, the MRTK ButtonCollection toolbar is modified by adding buttons with the following customized function:

- “Set Layer”: change the vertical layer onto which product components are placed;
- “Reset Configuration”: delete all product components thus resetting the configuration;
- “Create Product Tree”: create an abstract representation of the configured product (see Sec. 2.3) to be sent to production.

In terms of hardware, the XR configurator runs on an XR-device (Microsoft HoloLens 2). This allows to integrate body movements thanks to which customers are able to design their own products through an active interaction with the product components. Fig. 6b shows an image of XR user experience from the user perspective. Namely, a user after interacting with standard objects which, similarly to the CCAD configurator, can be placed and rotated on the assembly plate, commences production by pressing one of the four buttons provided by the interface.

#### Sustainability feedback

The goal of the configurator GUI as intended in this project, is not only to enable and assist with the configuration of a product to be assembled by other production modules, but also to highlight how the integration of intuitive but representative sustainability metrics could be possible and support decision-making e.g. opting for a lightweight product solution or one that minimizes the carbon emissions for the Cradle-to-Gate scenario. As visible in Fig. 6b, the XR configurator integrates the real-time feedback of a sustainability indicator directly related to the type of selected components and their number, further stressing how the appropriate infrastructure (as software and hardware) helps to include sustainability in production planning. In practice, the metrics chosen for the sustainability performance evaluation do not need to be complex but can be extremely intuitive and selected based on the company priorities and/or sensibility considerations e.g., to favour aspects such as Carbon Footprint for high-energy intensive industrial activity, water consumption for food processing or paper manufacturing, or material weight for situations in which transportation and logistics play a key role in decision making.

**Figure 7:**
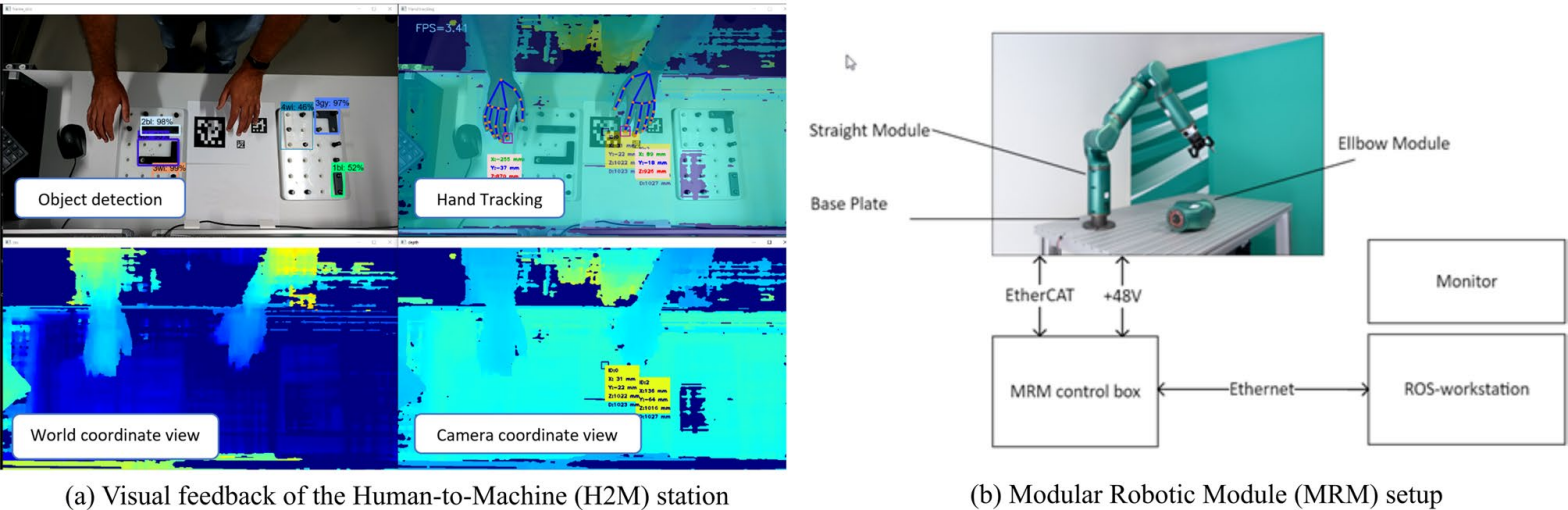
Details of the robotics assembly modules.

### Human-to-machine transfer

Thanks to multiple development iterations on a dedicated hardware setup, we explored pros and cons of different machine learning approaches for knowledge transfer as well as benefits and drawbacks of traditional marker-based motion tracking systems. The H2M module builds upon a standard Bosch Rexroth ActiveAssist height-adaptable industrial table with a superstructure for fixing trays and illumination, customly equipped with two Luxonis OAK-D Stereo smart cameras^52^. The latter are exploited for marker-free human-hand tracking implemented using mediapipe^53^ running on the in-camera Myriad-X AI accelerator. The choice of a marker-free solution was motivated to be as least invasive as possible for users, while mediapipe was selected for its broad usage in real world applications, its long term support and the possibility to run it “in-camera”. The extracted hand coordinates are transformed to a common world coordinate system, postprocessed, merged and provided over an API to other modules, see Fig. 7a. The video stream of the cameras is also used for part recognition using a mobilenet-v2 based Convolutional Neural Network^54^. Additionally, the assembly station is equipped with an USB foot-pedal, allowing the operator to manually insert events into the data stream provided to other stations (currently denoting, but not limited to, start and end of a part manipulation). Also, the station consists of several custom developed Python application programs communicating with each other through JSON encoded data over ZeroMQ^55^. Finally, by recalling that the final aim of the station is to use recorded trajectories to extract knowledge to be transferred to the robotic assembly module, the station implements a machine learning program based on Gaussian Mixture modeling (GMM) and regression^56^. GMM was chosen among other approaches because of (1) being data efficient, e.g., with respect to artificial neural network; (2) requiring little prior knowledge; (iii) falling onto the class of unsupervised learning approaches thus not requiring labeling during data collection.

### Assembly and production

For assembly and production, two separate modules are implemented, one using a fixed-structure robot; the other exploiting a modular reconfigurable robot (MRM). In particular, the former is used to implement classical control approaches; conversely, the MRM is used to explore recent human-to-machine transfer methods^43,44,57^ as well as approaches for multi-objective optimization of the robot configuration^46^. For both modules, the selection of ROS as middleware enabled us to leverage on out-of-the-box robot-manipulation, perception, control, visualization and learning tools and to accelerate therefore the development processes.

Hardware-wise, we adopt a reconfigurable platform by *Alberobotics*, a Technology Transfer Project of the Italian Institute of Technology (IIT), specifically developed, and thus selected, to allow for (1) easy and fast assembly of the system, (2) variable size passive link modules, (3) support of Robot Operating System (ROS) API, (4) low-level access to joint actuators, (5) availability of a simulation suite and, (6) joints communication through EtherCAT-field bus. As shown in Fig. 7b, the platform consists of three active straight joints, three active elbow joints, the control-box, a ROS-workstation, and a monitor. Software-wise, to interface the MRM and the H2M modules, the robotic platform has been enhanced with a custom-developed ROS-based toolchain used to optimize the robot composition for specific tasks, e.g. according to sustainability objectives. Finally, for the fixed-structure robotic assembly module, we make use of a Franka Emika Panda 7-DoF robot controlled (similarly to the MRM) using the available ROS-interface.

## Discussion and conclusion

This work presents a realization of an in-lab sustainable and flexible manufacturing system. The work addresses sustainability and flexibility at different productions levels in order to exploit their benefits from a holistic perspective. In particular, our developments contribute to advancing the state of the art addressing four main challenges: (1) supporting tools to explore the potential of I4.0 towards sustainable production; (2) managing the configurability and customization possibilities of products; (3) effectively handling the flexibility provided by a production system with rapid reconfiguration capabilities; (4) integrating hardware and software flexibility by using reconfigurable robotics and machine learning methods. The results have been integrated into the physical demonstrator (located in Fraunhofer Italia Research ARENA). To the best of the authors knowledge, the demonstrator represents a unique effort towards a complete small scale production system which can be used to showcase sustainability and reconfigurability at different levels of a production system.

We argue that, despite the demonstrator being implemented in a in-lab environment, the transfer and application of the proposed methods to a real world setup is already possible. Indeed, most of the technologies used in our implementations are off-the-shelf commercial solutions. In particular, the Smart Configurator (sections “Product personalization for flexible and sustainable production” and “Product configurator(s)”) is based hardware-wise on Microsoft HoloLens 2 and software-wise on Rhinoceros 3D^49^, Unity^50^ and the MRTK^51^. The Human-to-Machine module (sections “Reconfigurable and learning modules for flexible and sustainable production processes” and “Human-to-machine transfer”) leverages, hardware-wise, two Luxonis OAK-D smart cameras^52^ in combination with open source mediapipe^53^ software library. Different is the Modular Robotics Module (sections “Reconfigurable and learning modules for flexible and sustainable production processes” and “Assembly and production”). While the software is based on the open source ROS, the hardware currently uses a prototype product from Alberobotics, but is accessed via industry standard communication protocols. The more critical component is represented by the MAS (section “Planning and control of flexible and sustainable production processes”) and, in particular, its interfaces. Indeed, the pure MAS software exploits open source libraries and does not require any specific hardware. However, each production machine to be exposed to the system requires the implementation of a dedicated *agent* fulfilling all the necessary communication and control interfaces. And, despite most existing machinery can be retrofitted using, e.g., additional (in our case Arduino) microcontrollers, this might not always be the case. Finally, in addition to what already discussed at the end of section “Product configurator(s)”, it is worth stressing that the connection with and integration of sustainability metrics can take different forms, from simpler read-only values (as in our current SPCS implementation) to proactive configuration suggestions delivered through the configurator GUI. At first instance, data like resources (power, water, material, etc.) consumption can naturally come from production modules. Either directly from machine APIs or from estimated models implemented within the software agents participating in the MAS as for the power consumed by our MRM. However, additional data can also be integrated from MPSs, ERPs or even external databases.

Looking ahead, the physical demonstrator of a small scale complete manufacturing system enables large potentials for future developments both at module as well as system level. Regarding the smart product configuration systems (section “Product personalization for flexible and sustainable production”) we are planning on the one hand to improve the user experience; on the other to integrate more sustainability feedback in order to proactively drive user’s choices. Regarding planning and control (section “Planning and control of flexible and sustainable production processes”) we are planning, on the one hand, to reinforce the demonstrator capabilities with additional production modules e.g., for handling the storage of production components; on the other, to implement and test different scheduling and multi-objective optimization approaches. Finally, regarding the learning and robotic modules (section “Reconfigurable and learning modules for flexible and sustainable production processes”) we are planning to include machine vision based capabilities for assembly tasks.

## Data Availability

Does not apply as permanent data have not being generated. For any information please contact the corresponding author.
